# Supplementary material for: Resilience of S309 and AZD7442 monoclonal antibody treatments against infection by SARS-CoV-2 Omicron lineage strains
Source: Nat Commun. 2022 Jul 2;13:3824. doi: 10.1038/s41467-022-31615-7 (PMC9250508; doi:10.1038/s41467-022-31615-7)
Supplement: Supplementary file 1 — Supplementary information [file 41467_2022_31615_MOESM1_ESM.pdf]

**SUPPLEMENTARY INFORMATION**

# Resilience of S309 and AZD7442 monoclonal antibody treatments against infection by SARS-CoV-2 Omicron lineage strains

***J.B. Case et al.***

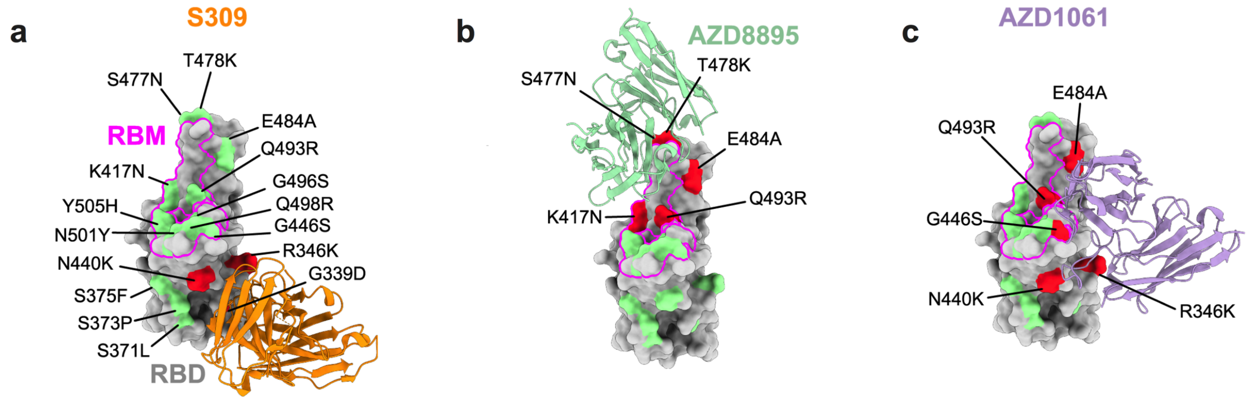


**Supplementary Figure 1. BA.1.1 spike protein substitutions and mAb epitopes**. Mutated residues in the BA.1.1 RBD relative to WA1/2020 are indicated in green in all three panels. The BA.1.1 RBD bound by mAbs S309 (orange, PDB: 6WPS) (**a**), AZD8895 (pale green, PDB: 7L7D) (**b**), and AZD1061 (purple, PDB:7L7E) (**c**) are shown. BA.1.1 substitutions in the respective epitopes of each mAb are shaded red, whereas those outside the epitope are shaded green. Structural analysis and depictions were generated using UCSF ChimeraX v1.3[44].

**a**

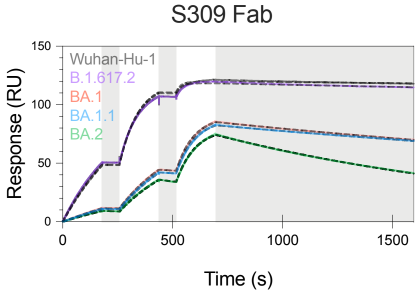


| S309 Fab | | | | |
|---|---|---|---|---|
| RBD variant | ka (1/Ms) | kd (1/s) | KD (M) | Avg. FC in KD vs. Wuhan-Hu-1 |
| Wuhan-Hu-1 | 1.39E+05 | 2.34E-05 | 1.71E-10 | 1.00 |
| BA.1 | 3.16E+04 | 3.03E-04 | 9.66E-09 | 56.63 |
| BA.2 | 3.12E+04 | 9.40E-04 | 3.18E-08 | 186.22 |

**b**

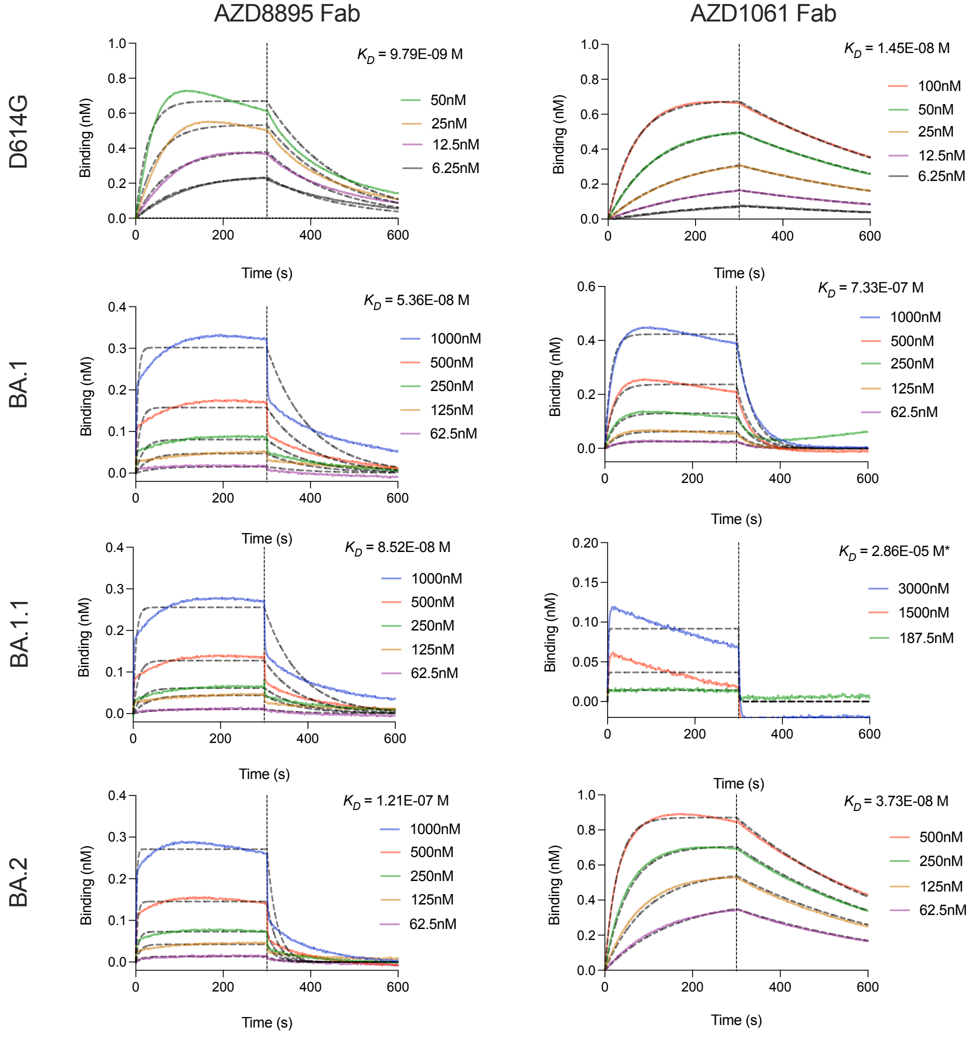


| | AZD8895 Fab | | | | AZD1061 Fab | | | |
|---|---|---|---|---|---|---|---|---|
| RBD variant | ka (1/Ms) | kd (1/s) | KD (M) | Avg. FC in KD vs. D614G | ka (1/Ms) | kd (1/s) | KD (M) | Avg. FC in KD vs. D614G |
| D614G | 6.16E+06 | 6.03E-03 | 9.79E-09 | 1.00 | 1.49E+05 | 2.17E-03 | 1.45E-08 | 1.00 |
| BA.1 | 1.90E+05 | 1.02E-02 | 5.36E-08 | 5.47 | 4.11E+04 | 3.01E-02 | 7.33E-07 | 50.55 |
| BA.1.1 | 1.44E+05 | 1.23E-02 | 8.52E-08 | 8.70 | 2.81E+04 | 8.02E-01 | 2.86E-05* | 1972.41 |
| BA.2 | 3.82E+05 | 4.61E-02 | 1.21E-07 | 12.36 | 6.51E+04 | 2.43E-03 | 3.73E-08 | 2.57 |

**Supplementary Figure 2. Binding affinities of S309 and AZD7442 Fab fragments against Omicron variant strains. a,** Single-cycle kinetics surface plasmon resonance (SPR) analysis of S309 Fab binding to the indicated SARS-CoV-2 RBD variants. S309 Fab was injected successively at 36, 143, and 571 nM. Dashed black curves show fits to a 1:1 binding model. White and grey shaded regions indicate association and dissociation phases, respectively. RU, response units; $K_D$, dissociation constant. The fold change (FC) is calculated relative to the affinity of Wuhan-Hu-1 RBD measured in parallel. **b**, Recombinant RBDs of the indicated SARS-CoV-2 variants were loaded onto biolayer interferometry (BLI) pins at a concentration of 5 µg/mL and the indicated concentrations of AZD8895 or AZD1061 Fabs were allowed to associate and dissociate for 300 s each step. Dashed black curves show fits to a 1:1 binding model. $K_D$, dissociation constant. The fold change (FC) is calculated relative to the affinity of WA1/2020 D614G RBD measured in parallel. Source data are provided as a Source Data file.

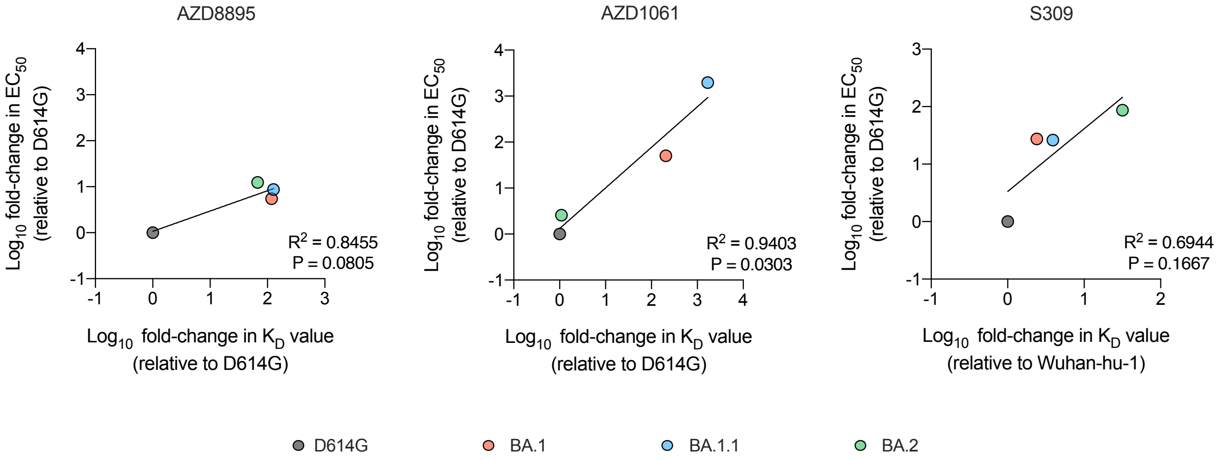


**Supplementary Figure 3. Correlation of binding affinity with neutralization activity.** Correlation analysis. The fold-change in $EC_{50}$ value obtained in **Fig. 1f-o** is plotted on the y-axis and the fold-change in variant RBD binding affinity obtained in **Supplementary Fig. 2** is plotted on the x-axis for each indicated mAb. Fold-changes were calculated relative to WA1/2020 D614G or Wuhan-Hu-1 as indicated. Best-fit lines were calculated using a simple linear regression. Two-tailed Pearson correlation was used to calculate the $R^2$ and P values indicated within each panel.

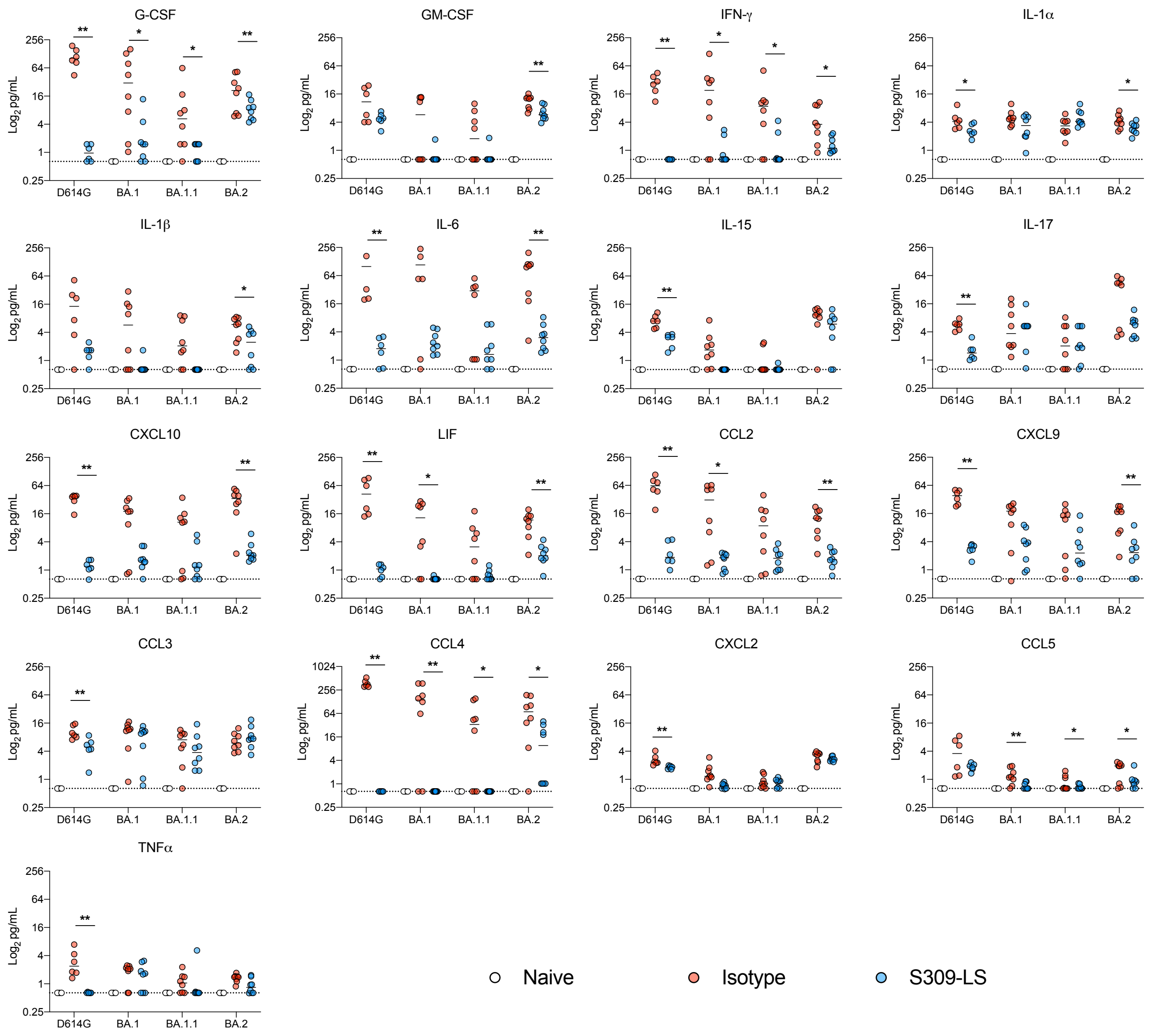


**Supplementary Figure 4. Cytokine and chemokine induction after S309-LS treatment and SARS-CoV-2 infection.** Individual graphs of cytokine and chemokine protein levels in the lungs of S309-LS mAb-treated K18-hACE2 mice at 6 (BA.2) or 7 dpi (all other strains) with the indicated SARS-CoV-2 strain (line indicates median value; n = 3, naïve; n = 6, D614G; n = 8, BA.1, BA.1.1, and BA.2; two-tailed Mann-Whitney test with comparison between the isotype control and mAb: *, $P < 0.05$, **, $P < 0.01$, ***, $P < 0.001$).

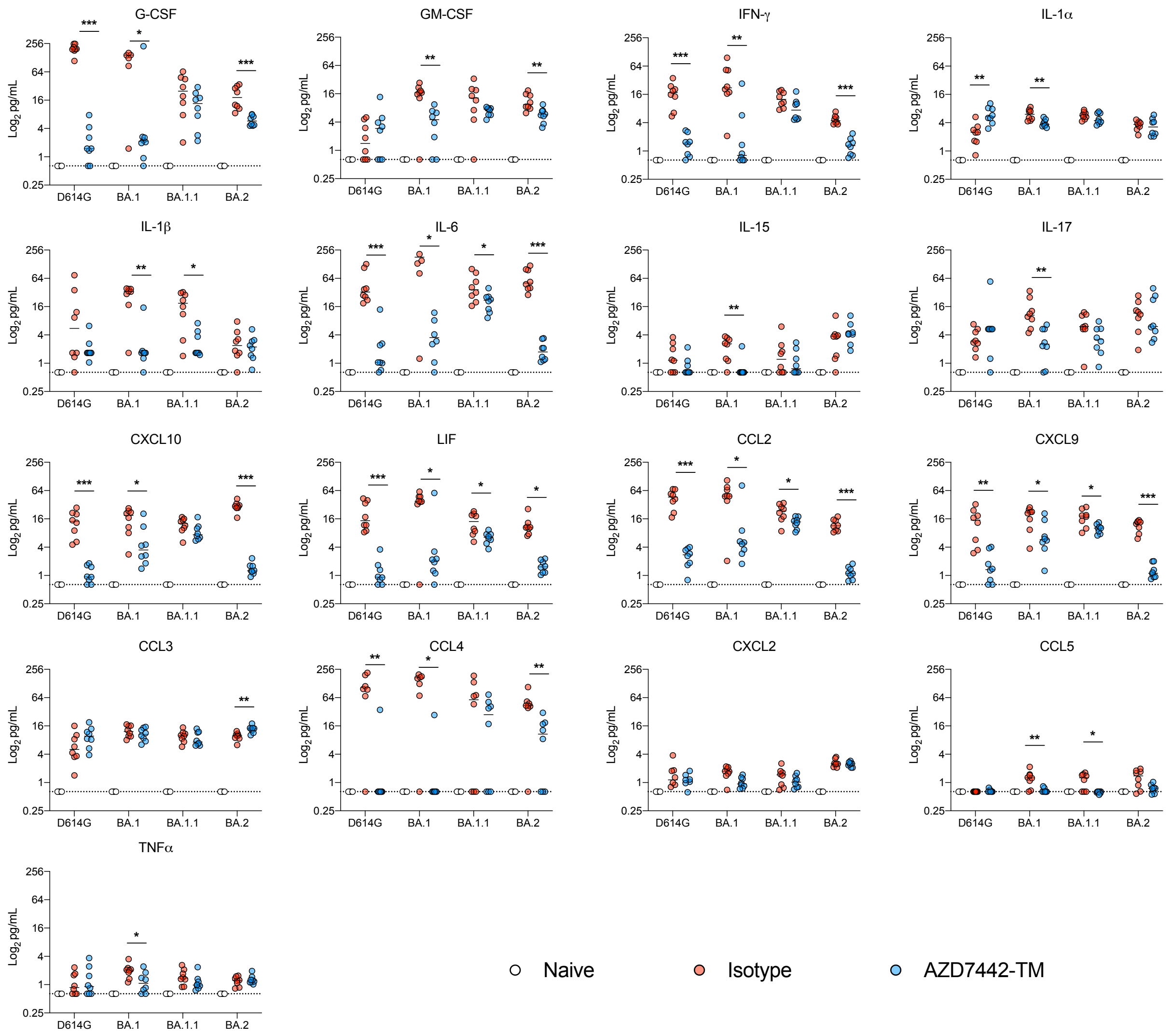


**Supplementary Figure 5. Cytokine and chemokine induction after AZD7442-TM treatment and SARS-CoV-2 infection.** Individual graphs of cytokine and chemokine protein levels in the lungs of AZD7442-TM mAb-treated K18-hACE2 mice at 6 (BA.2) or 7 dpi (all other strains) with the indicated SARS-CoV-2 strain (line indicates median value; n = 3 naive, n = 8 for all other groups; two-tailed Mann-Whitney test with comparison between the isotype control and mAb: *, $P < 0.05$, **, $P < 0.01$, ***, $P < 0.001$).

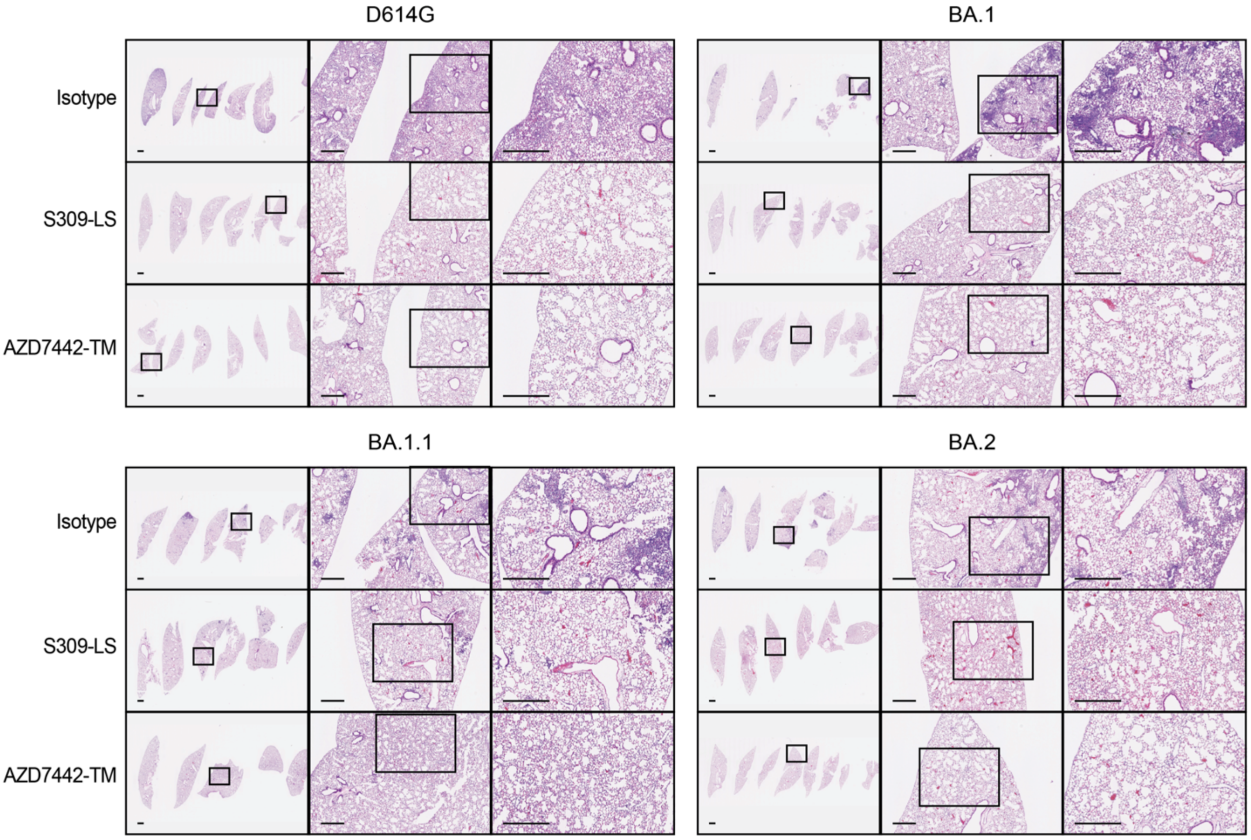


**Supplementary Figure 6. S309-LS and AZD7442-TM prevent Omicron variant-mediated lung pathology.** Hematoxylin and eosin staining of lung sections from eight-week-old female K18-hACE2 mice treated with 200 µg of the indicated mAb by intraperitoneal injection one day before intranasal inoculation with $10^3$ FFU of the indicated SARS-CoV-2 strain. Tissues were collected at six (BA.2) or seven days (all other strains) after inoculation. Images show low (left), medium (middle; boxed region from left), and high (right; boxed region from middle) power magnification. Scale bars indicate 10 mm, 250 µm, or 500 µm, respectively. Representative images are from three mice per group.

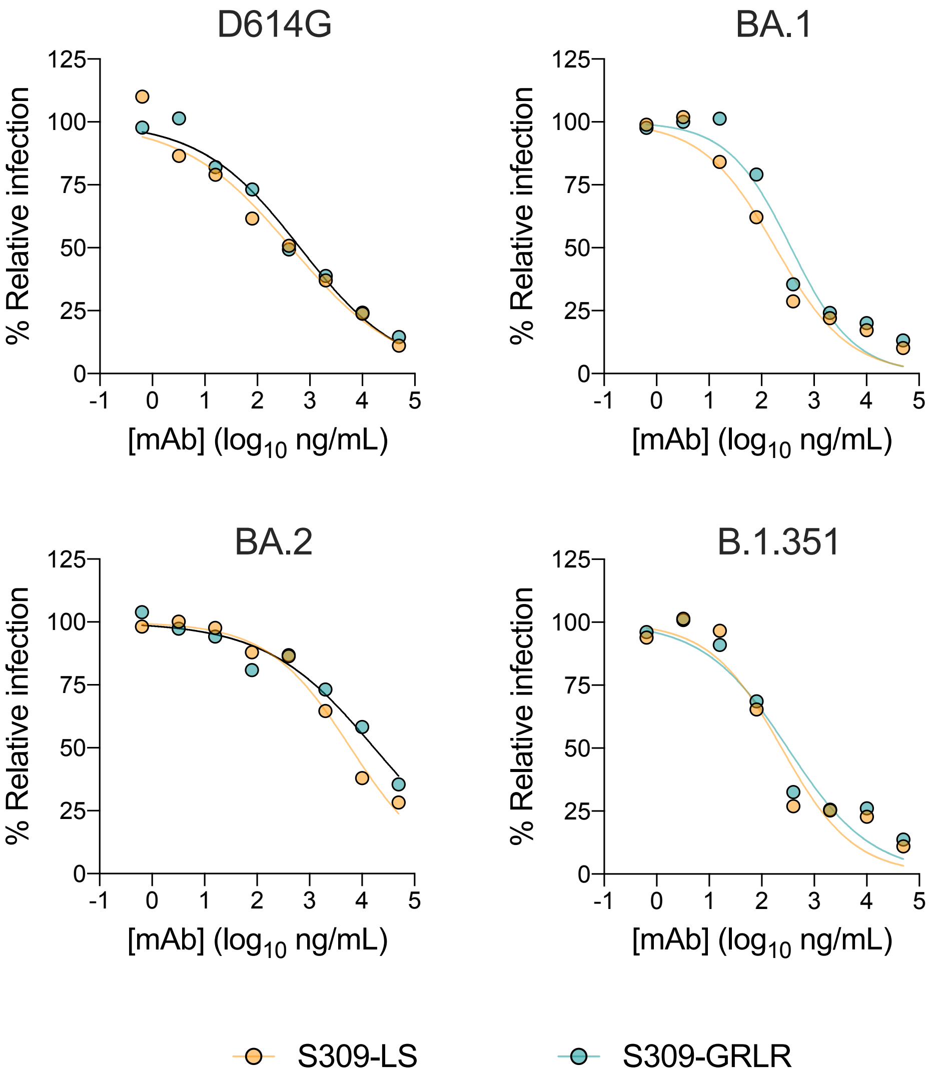


**Supplementary Figure 7. Neutralization of SARS-CoV-2 variants by S309-LS and S309-GRLR mAbs.** Neutralization curves in Vero-TMPRSS2 cells comparing infection of the indicated SARS-CoV-2 strain in the presence of each mAb. The average of two experiments performed in technical duplicate are shown. For D614G, BA.1, and BA.2 strains, the S309-LS neutralization data from **Fig. 1f** are shown for comparison. Source data are provided as a Source Data file.

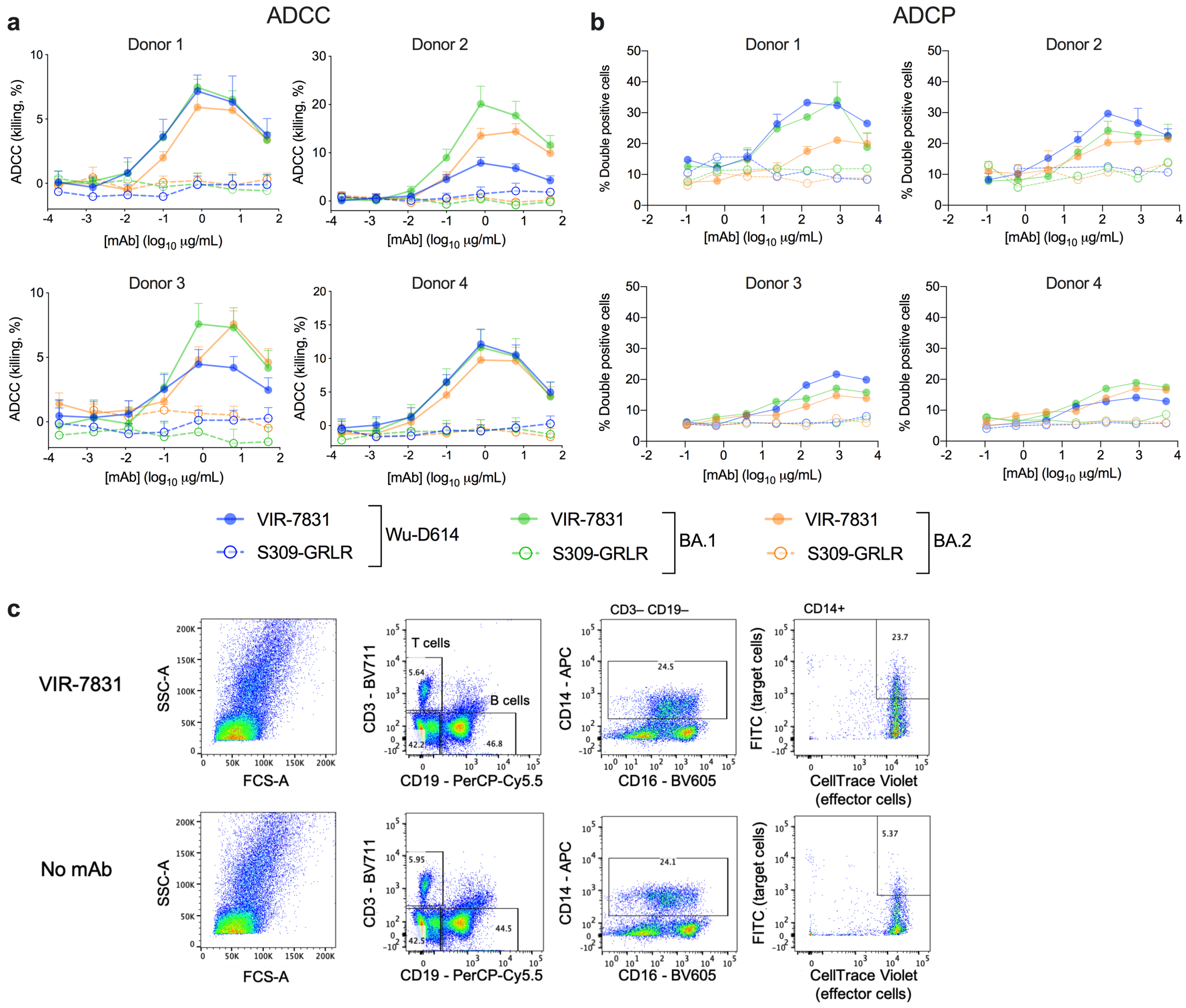


**Supplementary Figure 8. VIR-7831-mediated ADCC with NK cells and ADCP with monocytes. a**, ExpiCHO-S cells transiently transfected with expression plasmids encoding Wuhan D614, BA.1, or BA.2 spike proteins were incubated with the indicated concentrations of VIR-7831 or S309-GRLR and mixed with NK cells isolated from healthy donors at a ratio of 1:9 (target:effector). Target cell lysis was determined by a lactate dehydrogenase release assay. Data are presented as mean values $\pm$ standard deviations (SD) from four donors. Each panel is an individual donor. Donors 1 and 3 are heterozygous for F158 and V158 FcγRIIIa, whereas donors 2 and 4 are homozygous for V158. **b**, ExpiCHO-S cells transiently transfected with Wuhan-1 D614, BA.1, or BA.2 spike proteins and fluorescently labelled with PKH67 were incubated with the indicated concentrations of VIR-7831 or S309-GRLR mAb and mixed with PBMCs labelled with CellTrace Violet from healthy donors carrying different FcγRIIA and IIIA genotypes at a

ratio of 1:20 (target:PBMCs). Association of $CD14^+$ monocytes with spike-expressing target cells (ADCP) was determined by flow cytometry. Data are presented as mean values ± SD from four donors. Each panel is an individual donor. **c**, From PBMCs, monocytes were gated as $CD3^-$ $CD19^-$ $CD14^+$ cells. For ADCP, % $FITC^+$ CellTrace $Violet^+$ $CD14^+$ monocytes were gated as indicated. The gate of positive cells was set based on the no mAb control.

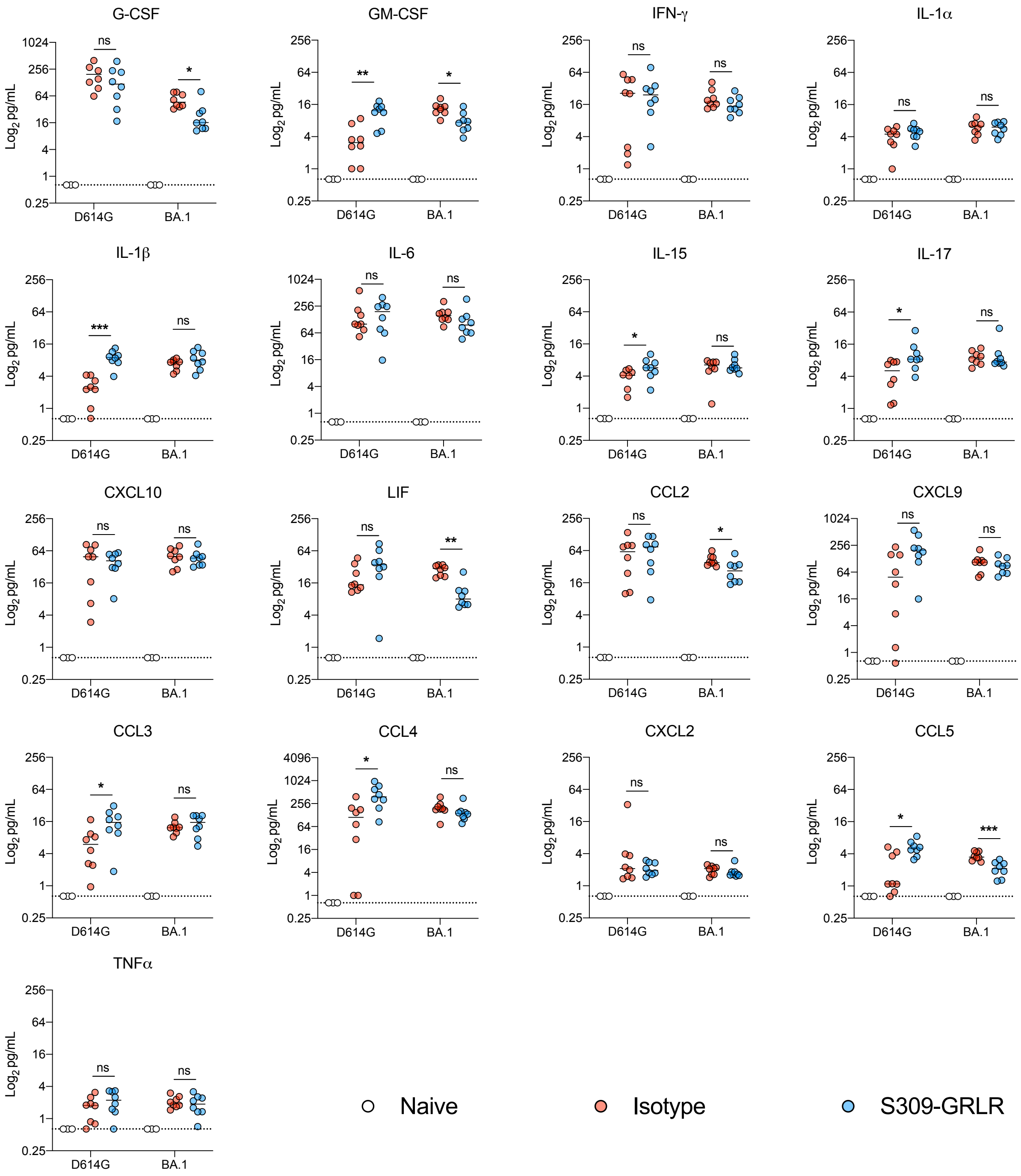


**Supplementary Figure 9. Cytokine and chemokine levels after S309-GRLR treatment and SARS-CoV-2 infection.** Individual graphs of cytokine and chemokine protein levels in the lungs of S309-GRLR mAb-treated K18-hACE2 mice at 7 dpi with the indicated SARS-CoV-2 strain (line indicates median; n = 3 naive, n = 8 for all other groups; two-tailed Mann-Whitney test with comparison between the isotype control and mAb: ns, not significant; *, $P < 0.05$, ** $P < 0.01$, ***, $P < 0.001$).

**Supplementary Table 1. Fold-changes in lung viral RNA titers.**

| **D-1 prophylaxis, K18-hACE2 mice** | | | | |
|---|---|---|---|---|
| | | | | |
| | Fold-reduction in lung viral RNA copies compared to isotype control mAb treated | | | |
| **mAb** | **D614G** | **BA.1** | **BA.1.1** | **BA.2** |
| S309-LS | 1744802.6 | 182.0 | 39.0 | 742.7 |
| S309-GRLR | 6.5 | 0.9 | N.D. | 1.3 |
| AZD7442-TM | 492342.7 | 92.1 | 4.1 | 103663.8 |
| | | | | |
| **D-1 prophylaxis, hFcγR TG mice** | | | | |
| | | | | |
| | Fold-reduction in lung viral burden compared to isotype control mAb | | | |
| | Viral RNA copies | | Infectious viral titer | |
| **mAb** | **2 dpi** | **4 dpi** | **2 dpi** | **4 dpi** |
| S309-GRLR | 2.6 | 3.3 | 3.6 | 8.8 |
| S309-LS | 47.3 | 14.8 | 292.2 | 81.3 |
| | | | | |
| | | | | |
| **D+1 therapy in K18-hACE2 mice** | | | | |
| | | | | |
| | Fold-reduction in lung viral RNA copies compared to isotype control mAb treated | | | |
| **mAb** | **D614G** | **BA.1** | **BA.1.1** | **BA.2** |
| S309-LS | 29.7 | 132.4 | 52.9 | 113.8 |
| AZD7442-TM | 27.5 | 131.0 | 4.5 | 32.6 |

N.D., not determined

**Supplementary Table 2. Omicron variant strain mutations determined by next-generation sequencing**.

| | BA.1 (B.1.1.529)<br><br>hCoV-19/USA/WI-WSLH-221686/2021 | | BA.1.1 (B.1.1.529 + R346K)<br><br>hCoV-19/USA/HI-CDC-4359259-001/2021 | | BA.2<br><br>hCoV-19/Japan/UT-NCD1288-2N/2022 | |
|---|---|---|---|---|---|---|
| | A67V | | A67V | | T19I | |
| | Δ69-70 | | Δ69-70 | | L24S | |
| | T95I | | T95I | | Δ25-27 | |
| | G142D | | G142D | | G142D | |
| | Δ143-145 | | Δ143-145 | | V213G | |
| | Δ211 | | Δ211 | | G339D | |
| | L212I | | L212I | | S371F | |
| | insertion 214EPE | | insertion 214EPE | | S373P | |
| | G339D | | G339D | | S375F | |
| | S371L | | R346K | | T376A | |
| | S373P | | S371L | | D405N | |
| | S375F | | S373P | | R408S | |
| | K417N | | S375F | | K417N | |
| | N440K | | K417N | | N440K | |
| | G446S | | N440K | | S477N | |
| | S477N | | G446S | | T478K | |
| | T478K | | S477N | | E484A | |
| | E484A | | T478K | | Q493R | |
| | Q493R | | E484A | | Q498R | |
| | G496S | | Q493R | | N440K | |
| | Q498R | | G496S | | N501Y | |
| | N501Y | | Q498R | | Y505H | |
| | Y505H | | N501Y | | D614G | |
| | T547K | | Y505H | | H655Y | |
| | D614G | | T547K | | N679K | |
| | H655Y | | D614G | | P681H | |
| | N679K | | H655Y | | N764K | |
| | P681H | | N679K | | D796Y | |
| | N764K | | P681H | | Q954H | |
| | D796Y | | N764K | | N969K | |
| | N856K | | D796Y | | | |
| | Q954H | | N856K | | | |
| | N969K | | Q954H | | | |
| | L981F | | N969K | | | |
| | | | L981F | | | |
